# Electrophoretically Deposited Chitosan/Eudragit E 100/AgNPs Composite Coatings on Titanium Substrate as a Silver Release System

**DOI:** 10.3390/ma14164533

**Published:** 2021-08-12

**Authors:** Łukasz Pawłowski, Michał Bartmański, Aleksandra Mielewczyk-Gryń, Bartłomiej Michał Cieślik, Grzegorz Gajowiec, Andrzej Zieliński

**Affiliations:** 1 Faculty of Mechanical Engineering and Ship Technology, Gdańsk University of Technology, Narutowicza 11/12, 80-233 Gdańsk, Poland; michal.bartmanski@pg.edu.pl (M.B.); grzegorz.gajowiec@pg.edu.pl (G.G.); andrzej.zielinski@pg.edu.pl (A.Z.); 2 Faculty of Applied Physics and Mathematics and Advanced Materials Centre, Gdańsk University of Technology, Narutowicza 11/12, 80-233 Gdańsk, Poland; alegryn@pg.edu.pl; 3 Faculty of Chemistry, Gdańsk University of Technology, Narutowicza 11/12, 80-233 Gdańsk, Poland; bartlomiej.cieslik@pg.edu.pl

**Keywords:** chitosan, Eudragit, AgNPs, electrophoretic deposition, smart coatings

## Abstract

Due to the possibility of bacterial infections occurring around peri-implant tissues, it is necessary to provide implant coatings that release antibacterial substances. The scientific goal of this paper was to produce by electrophoretic deposition (EPD) a smart, chitosan/Eudragit E 100/silver nanoparticles (chit/EE100/AgNPs) composite coating on the surface of titanium grade 2 using different deposition parameters, such as the content of AgNPs, applied voltage, and time of deposition. The morphology, surface roughness, thickness, chemical and phase composition, wettability, mechanical properties, electrochemical properties, and silver release rate at different pH were investigated. Using lower values of deposition parameters, coatings with more homogeneous morphology were obtained. The prepared coatings were sensitive to the reduced pH environment.

## 1. Introduction

Postoperative bacterial infections are one of the most common reasons for unsuccessful implant procedures [1]. The formation of a biofilm, a coating composed of bacteria, fungi, and other microorganisms, which are resistant to the human immune system and antibiotic therapy, contributes to the development of bacterial infection and implant rejection by the body [2]. The development of bacterial infection following a dental implant placement usually leads to, for example, peri-implantitis, which is described as a destructive inflammatory process developing around the peri-implant tissues leading to bone resorption, deterioration of osteointegration, peri-implant pocket formation, and ultimately, bone loss [3]. It is estimated that in about 10% of implants, the peri-implantitis phenomenon occurs 5–10 years after implantation [4]. To prevent bacterial settling and biofilm formation on the implant surface, it is necessary to create coatings for the implants that will exhibit bactericidal properties [5]. There are many examples of such coatings in the literature; however, the problem is the controlled release of the therapeutic substance from the coating covering the implant throughout the implant’s lifetime [6,7,8,9]. Usually, the active substance is released immediately after the implant placement in the environment, simulating body fluids, which results in the burst release phenomenon, i.e., the release of a high dose of the drug in a short time; this may harm the human body [10].

Currently, active coatings are designed from a wide range of materials, mainly biopolymers, which form a system of controlled delivery of the medicinal substance to the perivascular tissues [11]. The features characterizing the inflammatory tissues, i.e., elevated temperature and lower pH value, are used for this purpose [12]. The base of such coatings is often so-called smart biopolymers, reacting to the external environment, e.g., temperature change, pH, UV-VIS radiation, or magnetic or electric field [13]. pH-sensitive biopolymers are polyelectrolytes containing weak acid or base groups that accept or donate protons when exposed to a changing surrounding environment. Changes in the pH may induce protonation or deprotonation of the functional groups of the biopolymer chain, leading to flocculation, change in the chain length, or homopolymer precipitation. Under the influence of environmental changes, it is also possible to self-organize the biopolymer into micelles, gel formation, or swelling [14]. Among the pH-sensitive biopolymers chitosan [15], Eudragit E 100 [16], poly(4-vinylpyridine) [17], and poly(L-histidine) are prominent [18]. They may form a matrix in which metallic nanoparticles with antibacterial properties can be dispersed.

Chitosan (chit) is one of the biopolymers most commonly used in controlled release systems. Apart from its application as a coating for implants, chitosan is used in the form of nanoparticles, microspheres, and hydrogels in bone tissue regeneration scaffolds, as a means for wound healing, or as a carrier of a therapeutic substance in drug delivery systems [19,20]. Chitosan coatings are prepared in combination with other biopolymers [21,22], bioceramics [23], and metallic nanoparticles [24]. The disadvantage is that chitosan is unstable at neutral pH and easily absorbs water by swelling, which results in the quick release of the drug [25,26]. The main problem of the systems mentioned above is the lack of control over the release of the medicinal substance. The burst release phenomenon often occurs; the active substance is released under conditions that are not characteristic of inflammatory conditions; thus, it is practically impossible to provide long-lasting protection against the development of bacterial infections of surrounding tissues. It is, therefore, necessary to limit the dissolution of the chitosan coating at neutral pH. Previous studies [27] confirmed that the addition of Eudragit E 100 (EE100), a cationic copolymer based on dimethylaminoethyl methacrylate, butyl methacrylate, and methyl methacrylate in a 2:1:1 ratio, to chitosan coatings significantly reduced the degree of degradation of these coatings in a simulated body fluid (SBF) in neutral pH while maintaining high sensitivity to the pH decrease.

Silver nanoparticles (AgNPs) show antibacterial and antifungal properties against a wide range of pathogenic microorganisms [28]. The mechanism of eradicating bacteria by silver is not fully explained. As a result of electrostatic action, silver causes changes in the structure of the bacterial cell membrane leading to its destruction. Moreover, due to the strong interaction of silver ions with thiol groups, important enzymes of the bacterial cells may be inactivated. The interaction of silver ions with the bacterial cell DNA results in the loss of its ability to replicate [29]. Silver nanoparticles in combination with a chitosan coating may provide a synergic antibacterial effect [30]. Moreover, the addition of silver nanoparticles to coatings based on polysaccharides improves their stability and increases their mechanical strength [31]. In addition, chitosan possesses the ability to form bonds with metal ions through electrostatic interaction with its electron-rich amino groups, increasing the stability of nanoparticles [32].

The aim of this research is the deposition of chit/EE100/AgNPs composite coatings by electrophoretic method and determination of the kinetics of the AgNPs release to the simulated body fluids at different pH values. The important process determinants for creating such composite coatings with EE100 compound by electrophoretic method on the surface of Ti grade 2 have not yet been determined, and no reports concerning investigations of coatings composed of chitosan/EE100 and silver nanoparticles have been found.

## 2. Materials and Methods

### 2.1. Preparation of Samples

Grade 2 titanium, delivered by the EkspresStal, Luboń, Poland, was chosen as a substrate (chemical composition according to the delivering company is illustrated by Table 1). Each sample cut from the bar (12 mm in diameter and 4 mm in height) was polished with SiC abrasive papers (Struers Company, Krakow, Poland) using a grinding device (Saphir 330, ATM GmbH, Mammelzen, Germany) and No. 800 sandpaper as the last. Before deposition, the samples were washed up with isopropanol (POCH S.A., Gliwice, Poland, 99.9%) and then with distilled water.

### 2.2. Preparation of Chitosan/Eudragit E 100/AgNPs Coatings

The suspensions prepared for tests contained different amounts of silver nanoparticles (delivered by Hongwu International Group Ltd., Guangzhou, China, mean size 30 nm), namely, 0.005 g (suspension A) and 0.01 g (suspension B); 0.1 g of chitosan (delivered by Sigma-Aldrich, St. Louis, MO, USA, high purity > 99%, MW ~310–375 kDa); 0.25 g of Eudragit E 100 (Evonik Industries, Darmstadt, Germany, purity 99.9%, MW ∼47 kDa); 0.1 mL of Polysorbate 20 (Tween 20) (Sigma Aldrich, St. Louis, MO, USA) applied as an effective dispersant of metallic nanoparticles, as shown previously [27,33]. All components were put in 100 mL of 1 vol.% acetic acid (Stanlab, Lublin, Poland, 99.9%) to dissolve. At first, the appropriate amounts of biopolymers (chitosan and Eudragit) were dissolved in an aqueous solution of acetic acid and stirred for 24 h at room temperature, using a magnetic stirrer (Dragon Lab MS-H-Pro+, Schiltigheim, France). Then, 1 hour before the deposition, metallic nanoparticles were introduced together with Polysorbate into the suspensions.

Different constant voltage values and deposition times were applied during electrophoretic deposition carried out at room temperature. Table 2 shows the designations of the samples with the relevant process parameters. The electrical network included the Ti grade 2 as a cathode and a platinum mesh as a counter electrode, and a power source (MCP/SPN110-01C, Shanghai MCP Corp., Shanghai, China). The distance between the electrodes was fixed for 10 mm. Following the deposition, the samples were washed with distilled water and dried in the air.

### 2.3. Testing of the Microstructure and Morphology of the Coatings

A high-resolution scanning electron microscopy (SEM, JEOL JSM-7800 F, JEOL Ltd., Tokyo, Japan) using 5 kV acceleration voltage was employed to determine the morphology of the composite coatings. Before SEM imaging, a 10 nm gold film was deposited on the surface of the specimen with a magnetron sputter (table-top DC, EM SCD 500, Leica, Wetzlar, Austria) operating under argon-inert atmosphere (Argon, Air Products, Warsaw, Poland, 99.999%).

The surface topography was observed with an atomic force microscope (NaniteAFM, Nanosurf AG, Liestal, Switzerland). A non-contact mode of testing was applied at a 55 mN force. The tests were made on a surface of dimensions 50 × 50 μm, and the arithmetic average deviation (Sa), the maximum peak height (Sp), and valley depth (Sv) were determined for the characterization of the surface topography. The thicknesses of the coatings were measured with a dual scope FMP10-20 coating thickness meter (SN100146594, Helmut Fischer GmbH, Sindelfingen, Germany) as means of 10 measurements for each sample.

The energy-dispersive X-ray spectroscopy (EDS, Edax Inc., Pleasanton, CA, USA) allowed a qualitative analysis of the chemical composition of the deposited coatings.

The phase composition of the prepared composite coatings was detected with the XRD spectroscopy (Philips X’Pert Pro, Almelo, The Netherlands) using 0.02 step and 2 s/point (Cu Kα, λ = 0.1554 nm) in 10–90° of the 2θ range at ambient temperature and atmospheric pressure.

The kind of chemical bonds forming between the biopolymers and nanoparticles was recognized by the Fourier-transform infrared spectroscopy (FTIR) with a spectrophotometer (Perkin Elmer Frontier, Waltham, MA, USA) in the range of 400–4000 cm^−1^ and at a 2 cm^−1^ resolution. The tests were undertaken in Attenuated Internal Reflection Spectroscopy (ATR) mode on as-prepared coatings on titanium grade 2 substrates.

### 2.4. Wettability Studies

The measurements of the water contact angle were carried out with a goniometer (Attention Theta Life, Biolin Scientific, Espoo, Finland) by the falling drop method and at room temperature. The distilled water drop volume was about 2 µL. Each test was completed in 10 s after drop out. Six measurements were taken for each sample, and their means were calculated.

### 2.5. Adhesion Studies

Adhesion of the coatings to titanium substrate was determined by scratch test (NanoTest Vantage, Micro Materials, Wrexham, UK). For each sample, 10 scratches were made with a length of 500 µm, increasing the load in the range of 0–200 mN and at a 1.3 mN/s load rate. For each measurement, from the observed rapid change in frictional force observed in the normal force and frictional force relationship, the force causing the total delamination of the coating from the surface was determined. All scratches were thoroughly examined with a light microscope (BX51, OLYMPUS, Tokyo, Japan).

### 2.6. Corrosion Studies

The Ti grade 2 substrate and coated samples used as tested electrodes were immersed in a chosen simulated body fluid (SBF of composition by the PN-EN ISO 10993-15 standard [34]) by dissolution in deionized water of the following substances: 0.13 gL^−1^ of (NH_2_)_2_CO, 0.33 gL^−1^ of KSCN, 1.5 gL^−1^ of NaHCO_3_, 0.26 gL^−1^ of Na_2_HPO_4_, 0.7 gL^−1^ of NaCl, 0.2 gL^−1^ of K_2_HPO_4_, and 1.2 gL−1 of KCl in 1 L of deionized water. The tests were conducted at 37 °C with a potentiostat/galvanostat (Atlas 0531, Atlas Sollich, Rębiechowo, Poland). The electric network was composed of Pt, a counter-electrode, and a silver chloride reference electrode. To achieve a steady open circuit potential (OCP), samples were stabilized in solution for 10 min before the experiment.

Subsequently, the electrochemical impedance spectroscopy (EIS) (Atlas 0531, Atlas Sollich, Gdańsk, Poland) investigations were performed with the same equipment as described above. The applied frequency ranged from 0.1 Hz to 100 kHz, and the amplitude was 10 mV. The acquired data were treated with the ZView software (Scribner Associates, Southern Pines, NC, USA) based on the equivalent electrical circuit.

Afterward, using a potentiodynamic mode, the polarization curves were determined. The tests were carried out within a scan range of −1.0/1.0 V at a 1 mV/s potential change rate. Employing the Tafel extrapolation approach, the corrosion potential (E_corr_) and corrosion current density (i_corr_) values were established based on obtained curves.

### 2.7. Silver Release Study

The silver release into the mentioned above SBF solution was analyzed with a microwave plasma atomic emission spectrometer (4210 MP-AES, Agilent, Santa Clara, CA, USA). The coated samples (A1 and B1) were immersed in 50 mL of SBF for 1 day. SBF solutions with different pH (3, 5, and 7) were prepared, their pH adjusted with HCl addition (30%, POCH, Gliwice, Poland) for simulating bacterial infection of peri-implant tissues [12,35]. The tests were carried out at 39 °C temperature, and the calibration solution was of the ICP grade. Four repetitions and two separate procedures made at different wavelengths were performed for different nanosilver contents. The wavelengths used were as follows: the 328.06 nm and 338.28 nm wavelengths were applied. The uncertainties were presented as Combined Standard Uncertainty (CSU) for all eight measurements based on the calibration solution.

## 3. Results and Discussion

### 3.1. Structure and Morphology of Chitosan/Eudragit E 100/AgNPs Coatings

**Figure 1:**
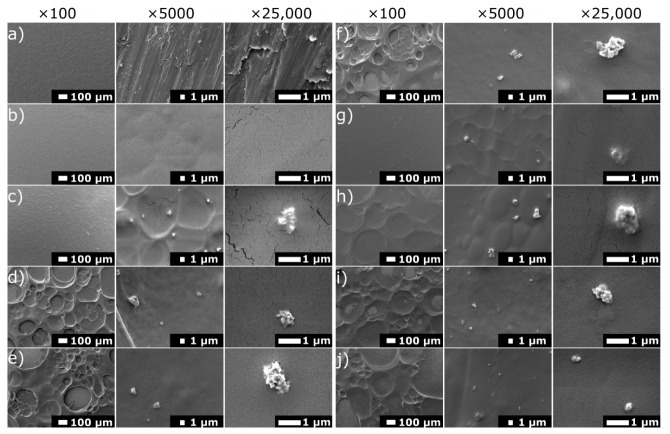
SEM images of the surface of (**a**) titanium substrate, (**b**) chit/EE100 coating, and the chit/EE100/AgNPs coatings prepared with various process parameters: (**c**) A1, (**d**) A3, (**e**) A1′, (**f**) A3′, (**g**) B1, (**h**) B3, (**i**) B1′, (**j**) B3′ sample.

Figure 1 shows the results of SEM imaging of a titanium substrate, chit/EE100 coating, and a set of chit/EE100 coatings with AgNPs additives. The Ti grade 2 sample revealed the microstructure resulting from the wet grinding process [36]. The experimental conditions resulted in the deposition of coatings with a rough or porous morphology, typical of chitosan-based coatings obtained from aqueous suspensions [37]. The porosity of the coatings is particularly evident in Figure 1d–f,h–j obtained at ×100 magnification. It may be an advantage, as the porosity of implant coatings could promote in vivo cell growth [38]. The reduction of pH was responsible for the protonation of the amino groups of chitosan and Eudragit E 100, and thus for their gradual charge and dissolution [39]. The chit/EE100 sample, prepared for comparison purposes at the same deposition parameters as sample A1, was characterized by high homogeneity. The titanium substrate was entirely covered by the coating. Higher magnification images showed the microporous structure of the coating. For chit/EE100/AgNPs coatings, the obtained images revealed the influence of the applied process parameters on the morphology of the coatings. Both the increase in the applied voltage and in the deposition time resulted in the formation of a more heterogeneous surface due to more rapid deposition kinetics [40]. The high deposition voltage was assumed to increase the velocity of particle movement by causing turbulence in the suspension and preventing the formation of a compact coating [41]. These coatings showed traces of hydrogen bubbles, which presumably resulted from water electrolysis during the EPD process [42]. The adverse effects of the formation of hydrogen bubbles on the homogeneity of coatings could be diminished by decreasing water content in the EPD suspension [43]. In some areas, this process caused a total exposure of the titanium substrate to the test fluid. It may also be considered whether an excessive content of chitosan cannot be responsible for this effect. According to Zhang et al. [42], chitosan suspensions possess a relatively low surface tension; hence, the increasing content of chitosan in the suspension could enhance the formation of bubbles that adhere to the deposited coating, increasing its porosity [44]. The smoothest coatings, which fully covered the substrate surface, were obtained at the lowest deposition time and voltage value, as also previously observed [27]. Comparing the images in Figure 1b,c, it can be then concluded that the addition of Ag nanoparticles resulted in the formation of a more heterogeneous coating, likely because the AgNPs were able to disturb the migration of the biopolymer particles [41].

The AgNPs showed a tendency to form agglomerates with a diameter of about 1 µm. Such aggregation of silver nanoparticles may cause a significant reduction in their antibacterial activity [45]. Despite that, small nanometric AgNPs groups were also visible. The images obtained at larger magnifications disclosed micro-cracks that did not appear in the biopolymer coating but only in the gold-sputtered layer, following an earlier study [33].

**Figure 2:**
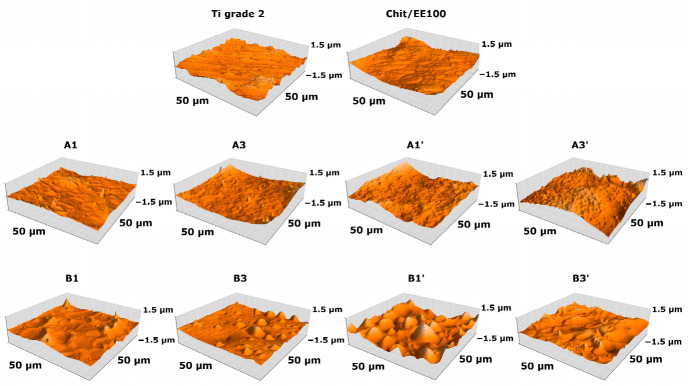
The surface topography of a bare titanium substrate, the chit/EE100 coating, and the set of chit/EE100/AgNPs coatings obtained by atomic force microscopy.

The surface topography of the bare titanium, the chit/EE100 coating, and the set of chit/EE100/AgNPs coatings observed by atomic force microscopy are presented in Figure 2. Quantitative parameters describing the surface topography and the values of thickness are listed in Table 3.

The Sa, Sp, and Sv values were calculated. The samples with coatings prepared at lower values of voltage and deposition time exhibited lower surface roughness compared to the bare Ti grade 2 substrate. As mentioned above, the increase in deposition voltage resulted in the more intensive water electrolysis and the formation of hydrogen bubbles on the cathode, which significantly decreased the homogeneity of the formed biopolymer coating, resulting in higher values of roughness parameters [40]. The addition of silver nanoparticles to the suspension increased the surface roughness of the deposited coatings. The increased surface roughness could promote bacterial cells’ adhesion and thus increase the risk of bacterial infection; therefore, the manufactured coatings were enriched with antimicrobial protection provided by AgNPs [46]. The AFM results obtained were in good correlation with the SEM images shown in Figure 1. The roughness parameters of implant coatings determine tissue adhesion, cell proliferation, and healing time [47]. High roughness enables greater tissue adherence and basic stabilization required between the implant and bone [48].

Thickness measurements of the produced coatings revealed an increase in coating thickness correlated with an increase in the deposition parameters used, i.e., time and voltage. The B-series coatings were characterized by relatively lower thickness. The higher amount of silver nanoparticles in the suspension most likely limited the migration of biopolymer particles, resulting in a thinner coating [41]. However, for the coatings deposited at lower parameters, the thicknesses of the A1 and B1 coatings were comparable to the thickness of the coating without silver nanoparticles. For implant applications, thicker coatings may improve the corrosion resistance of the implant, but the mechanical properties are reduced [49]. It is therefore necessary to select the process parameters of the EPD to obtain coatings of such a thickness as to achieve adequate corrosion protection while maintaining satisfactory mechanical properties. Higher standard deviations of the thickness were typical of more non-homogeneous coatings, which occurred at higher voltage and deposition time values.

**Figure 3:**
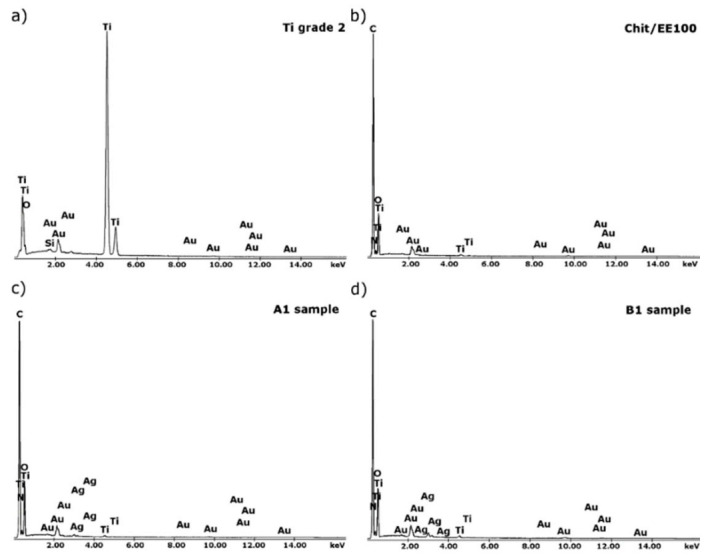
EDS spectra of (**a**) a bare titanium substrate; (**b**) the sample with the chit/EE100 coating and samples with chit/EE100/AgNPs coating: (**c**) A1 and (**d**) B1.

The results of EDS analysis for titanium substrate, chit/EE100 coating, and chit/EE100/AgNPs coatings (A1 and B1 samples) are shown in Figure 3. This qualitative analysis was performed with the samples prepared for the SEM examinations; therefore, the spectra contained peaks related to gold (Au). For the substrate, peaks related to titanium (Ti) and silicon (Si) were noted. The presence of silicon likely resulted from SiC abrasive paper. In the case of samples with biopolymer coatings, peaks referring to carbon (C), oxygen (O), and nitrogen (N) were recorded, accompanied by a decrease in the intensity of Ti peaks. In the case of chit/EE100/AgNPs samples (samples A1 and B1), EDS analysis confirmed the presence of silver (Ag) in the produced coatings and no contamination of the samples with other elements during the EPD process.

**Figure 4:**
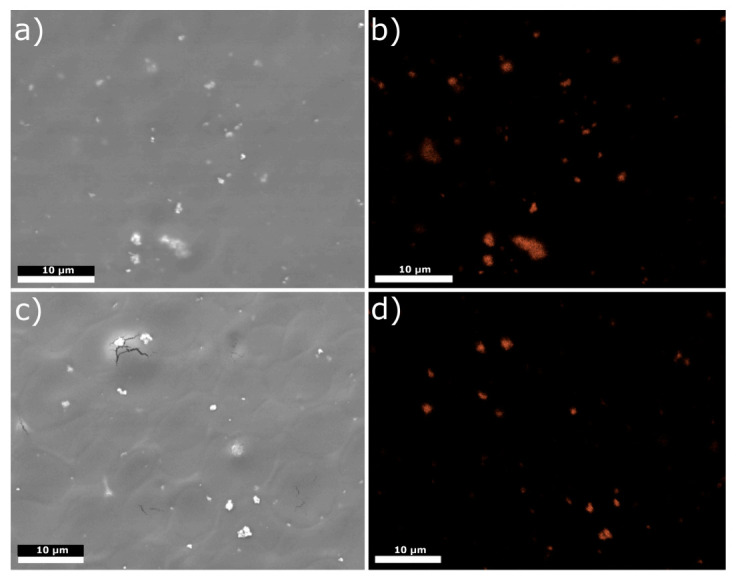
SEM images (**a**,**c**) and Ag distribution maps (**b**,**d**) of a selected area of (**a**,**b**) A1 and (**c**,**d**) B1 samples.

Figure 4 depicts silver distribution maps in selected areas of A1 and B1 samples. Silver nanoparticles were reasonably well-distributed in the coatings but revealed a tendency to form agglomerates of diameter above 1 µm despite the addition of dispersant to EPD suspension. Visible cracks in the coatings, as in Figure 4c, represent cracks in the sputtered gold layer. Agglomeration of silver nanoparticles decreases the release of silver ions and the antibacterial protection of coating as such agglomerates possess a lower surface-to-volume ratio and less possibility of interaction with bacteria as compared to isolated nanoparticles [33]. The problem of agglomeration could be solved by adding dispersants such as Polysorbate 20, which stabilizes AgNPs by intensifying existing electrostatic repulsion forces present among these particles. It may be considered whether in the present study, because of the demonstrated results, the amount of the dispersant was insufficient [45,50].

**Figure 5:**
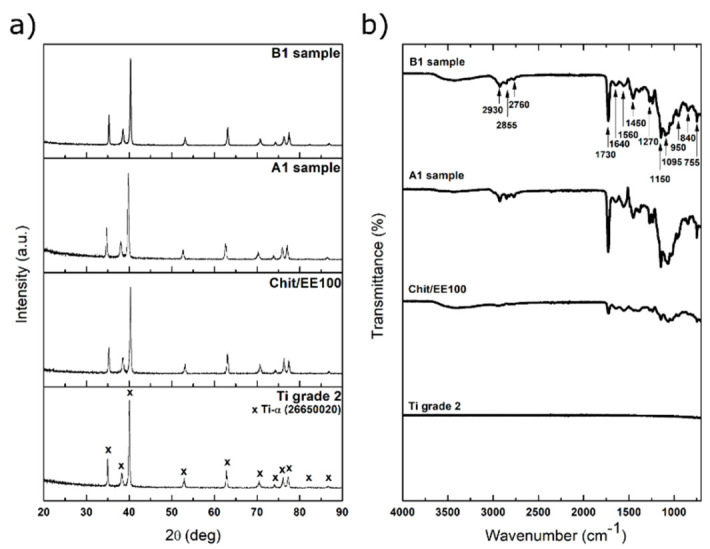
(**a**) X-ray diffractograms and (**b**) FTIR spectra of the bare titanium substrate, the sample with the chit/EE100 coating, and samples with chit/EE100/AgNPs coating (A1 and B1 samples).

X-ray diffractograms of a bare titanium substrate, chit-EE100 coating, and chit-EE100-AgNPs coatings (A1 and B1 samples) are demonstrated in Figure 5a. In all cases, only the peaks attributed to the alpha phase titanium were present (JCPDS file 44-1294). This may indicate the low thickness of the biopolymer coatings. Presumably, due to low concentration and nanocrystallinity, the peaks corresponding to silver are not distinguishable in the diffractograms [51]. Moreover, the main peak of the nanosilver (2θ = 38°, JCPDS file 04-0783) was overlapped with the others from the substrate material.

Figure 5b demonstrates the FTIR spectra of the investigated samples. Higher intensities were observed for samples with the AgNPs. In the range of 1300–1150 cm^−1^, typical bands of ester groups of EE100 appeared. In particular, a strong C=O ester stretching band was found at 1730 cm^−1^. The CH_x_ vibrations can be distinguished at 1385, 1450, and 2930 cm^−1^. The absorptions peaks at 2760 and 2855 cm^−1^ can be caused by the dimethylamine groups of EE100 [52]. The obtained spectra also show peaks characteristic of chitosan [53]. Stretching vibrations of OH groups can be observed in the 3750 cm^−1^–3000 cm^−1^ range, which coincided with those of C-H bonds in -CH_2_ and -CH_3_ groups. The bands in the range of 1640–1450 cm^−1^ can be attributed to carbonyl bonds (C=O) of the amide groups of chitosan, while the CO bond vibration is visible as the absorption in the range from 1150 to 1000 cm^−1^. Despite the small peaks of chitosan and Eudragit E 100 in the diffractograms, their presence in the coatings is detected by FTIR results, as shown before [27].

### 3.2. Wettability Studies

**Figure 6:**
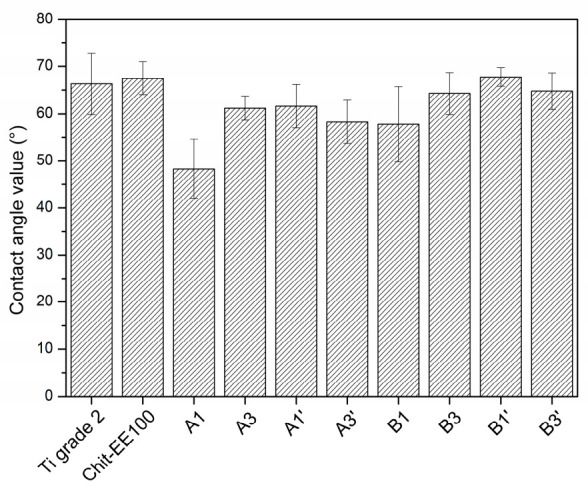
The results of water contact angle measurements for the bare titanium substrate, the chit/EE100 coating, and the chit/EE100/AgNPs coatings prepared with different process parameters; data are presented as the mean ± SD (*n* = 6).

Figure 6 depicts the results of contact angle measurements for the Ti grade 2 substrate, chit/EE100, and chit/EE100/AgNPs coatings. All samples showed hydrophilic properties (contact angle below 90°). The wettability of the chit/EE100 coating was similar to that of the titanium substrate. The addition of AgNPs to biopolymer coatings resulted in lowering the contact angle. An increase in AgNPs concentration in suspension (suspension B) additionally decreased the wettability of coatings; an upward trend in the measured contact angle values was observed. In addition, AgNPs agglomeration contributed to an increase in the hydrophobicity of the coating as compared to well-dispersed nanoparticles [54]. Therefore, more AgNPs agglomerates likely appeared in coatings prepared from suspension B (higher concentration of metallic nanoparticles), which resulted in a higher contact angle of these coatings. The obtained wettability results are in accordance with the measurements of surface roughness of the tested samples. For the chit/EE100/AgNPs coatings, a decrease in the wettability of the coating surface was observed as the surface roughness increased. The wettability of biomaterials is a significant parameter influencing protein adsorption, cell and bacterial adhesion, platelet adhesion, and blood coagulation [55]. An increase in the wettability of the biomaterial surface induces an improvement in cell proliferation, such as fibroblasts [56]. Concerning bone cells, the optimum contact angle range providing the best cell proliferation is assumed to be 35–85°; the recommended is 55° [57]. Thus, all of the samples tested in this study fit within this range.

### 3.3. Mechanical Studies

**Figure 7:**
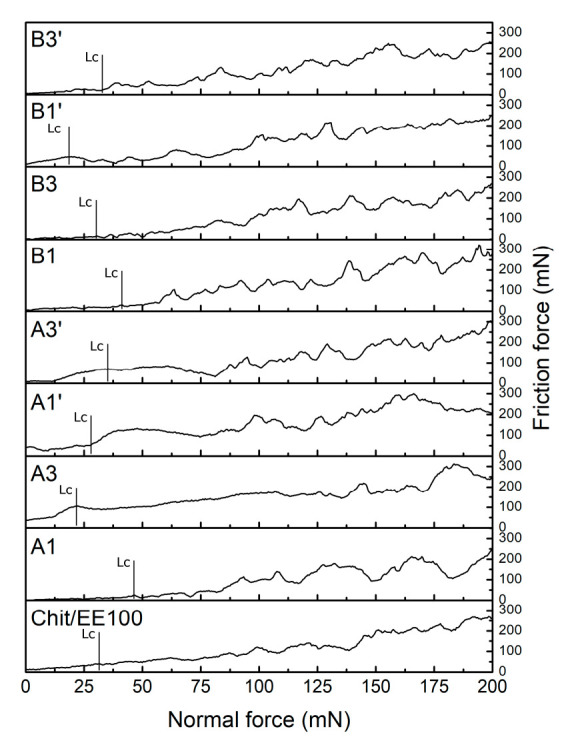
The friction force versus normal force dependence obtained for chit/EE100 and chit/EE100/AgNPs coatings along with an indication of the critical force (*Lc*) leading to complete delamination of the coating from the titanium substrate.

Proper adherence of the coating to the surface of the implant is essential because the implant is subjected to high loads, which may destroy the coating [58]. Figure 7 illustrates the friction force vs. normal force relationship and the critical force leading to the delamination of the coating. The critical force value was established on the basis of the above-mentioned curve and microscopic inspection of the created scratch. Table 4 collects the average values of the critical loading force (*Lc*) and the respective critical friction force (*Lf*) obtained in the scratch test.

The coatings deposited at lower process parameters (samples A1 and B1), i.e., time and voltage, were characterized by the highest adhesion. The deposition process parameters presumably resulted in this case in mild deposition kinetics for the biopolymers and nanoparticles, which resulted in a more packed and homogenous coating [59]. For chit/EE100 and A1 samples, the adhesion is comparable. However, when referring to earlier results [27], coatings containing silver nanoparticles revealed lower adhesion than chit/EE100 coatings. In the literature, there are almost no reports on adhesion to metal substrates of chit/EE100 coatings doped with nanometals. High values of standard deviations of *Lc* and *Lf* parameters indicate the heterogeneity of the coatings.

For coatings with poor mechanical properties and polymeric materials, it is difficult to determine coating cohesion forces. In the scratch test experiment, it was only possible to determine the complete delamination of the coating from the titanium substrate, and this was referred to as coating adhesion [60]. It is challenging to relate the obtained results to the actual mechanical loads exerted on the coating for proposed modifications such as dental implants. The difficulties are mainly due to the complex geometry of dental implants and the lack of a test technique that is able to study the adhesion of coatings on such surfaces. The technique used only indirectly correlates with the axial stresses present in the apparent implant materials [61,62].

### 3.4. Corrosion Studies

**Figure 8:**
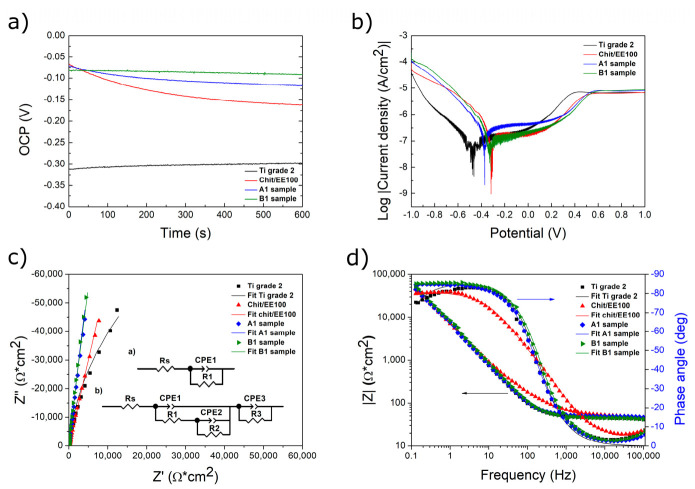
The results of electrochemical tests: (**a**) open circuit potential (OCP), (**b**) potentiodynamic polarization curves, and the experimental and simulated (**c**) Nyquist graphs with the equivalent circuit used to simulate experimental impedance data ((**a**)—for bare Ti grade 2 substrate, (**b**)—for prepared coatings), and (**d**) Bode-Z and Bode-phase graphs of the bare titanium substrate, the chit/EE100 coating, and chit/EE100/AgNPs coatings (A1 and B1 samples) immersed in the SBF at 37 °C.

Figure 8a presents the variation of the open circuit potential (OCP) over time for the investigated samples: Ti grade 2 substrate, chit/EE100 coating, and chit/EE100/AgNPs coatings (A1 and B1 samples) using the SBF solution as a corrosion medium at 37 °C. This analysis provides evidence of the stability of metallic compounds in the test fluid electrolyte in which they are immersed as well as indicates the corrosion potential [63].

All samples reached stability after about 600 s of immersion. A certain transition towards a positive potential value was observed for the Ti grade 2. The reason can be an appearance of a protective oxide layer on the surface in the literature [64]. In the case of the samples covered with coatings, a slight decrease in OCP value over time was observed. All coated samples showed stabilization of OCP potential between −0.2 and −0.05 V, and for the Ti grade 2 sample, about −0.3 V. The addition and increasing content of AgNPs in chit/EE100 coatings caused an increase in the OCP value. The most positive value of the potential was recorded for the B1 sample, most likely due to the presence of more unreduced silver ions in the coating richer in AgNPs agglomerates [65].

Potentiodynamic polarization curves obtained for all tested samples in the SBF solution are depicted in Figure 8b. Table 5 summarizes the open circuit potential (OCP) values and specific corrosion parameters determined based on Tafel extrapolation, i.e., E_corr_ and i_corr_.

The cathode branch of the polarization curves is associated with the release of hydrogen, while the anode branch is associated with the dissolution of the coating and substrate [66]. The E_corr_ values for coated samples were higher and shifted towards positive values compared to those of titanium substrate. They demonstrated lower corrosion current density values compared to the reference sample so that they are protective layers separating the environment and titanium base [59] and possess a higher corrosion resistance. The lowest corrosion current density value was appointed for the B1 sample. The addition of AgNPs caused only a slight increase in the corrosion resistance of chit/EE100 costings that may be attributed to the relatively inter behavior of noble silver nanoparticles [67]. The B1 sample contained the highest amount of silver; hence, it revealed the highest corrosion resistance.

Figure 8c,d illustrates the obtained EIS results, experimental and simulated. The experimental data were fitted using the equivalent electrical circuits as in Figure 8c, derived in other research work on similar biopolymer coatings doped with metallic nanoparticles [66]. To obtain the best fit of the experimental data, two equivalent circuits were proposed: for the bare substrate, insert (a), and for the coated titanium substrate, insert (b). Considering the roughness and unevenness of the bare sample surface as well as of the coatings, a constant phase element (CPE) was used instead of an ideal capacitor. The applied circuits consisted of R_s_—electrolyte resistance between the working and reference electrodes; R_1_ and CPE_1_—the barrier resistance and constant phase element of the native titanium oxide layer; R_2_, R_3_, and CPE_2_, CPE_3_, being, respectively, the electrical resistances and constant phase elements of the chit/EE100 and chit/EE100/AgNPs coatings. The formula for the CPE impedance was shown elsewhere [68].

Figure 8c represents the experimental and simulated Nyquist plots of Ti grade 2, chit/EE100, and chit/EE100/AgNPs coatings in the SBF. All plots were well defined at high and low frequencies. The obtained Nyquist graphs were similar to those presented previously for chitosan coatings with metallic nanoparticles [66]. Impedance was characterized by a quarter-round capacitive loop for all samples. The impedance of the coated samples was higher as compared to a bare substrate confirming the effect of chit/EE100 and chit/EE100/AgNPs coatings on the increase of the corrosion resistance of titanium. Moreover, the impedance was increased by the addition of silver nanoparticles, likely due to their chemical inertness and a low penetration rate of the solution into the coating [67].

According to the Bode-phase diagrams in Figure 8d, at high frequencies, the phase angles of the Ti grade 2 and chit/EE100/AgNPs samples were approaching 0°, indicating that the impedance was dominated by electrolyte resistance. In the case of chit/EE100 coated samples, the phase angle of the coated samples increased to about 10°, leading to the conclusion that the impedance was a capacitive reaction that was associated with the coating [66]. The phase angles of chit/EE100/AgNPs-coated samples were smaller than the phase angle of the chit/EE100-coated sample, which implied that the addition of Ag nanoparticles could impair the capacitive behavior of the chit/EE100 coating. For lower frequencies, the phase angles of uncoated and coated samples were related to the capacitive response of the titanium oxide layer, for which the phase angle changed due to the change in its polarity [40]. The phase angles of Ti grade 2 and chit/EE100/AgNPs samples were higher than those of chit/EE100.

Figure 8d also contains the Bode-Z diagrams of the tested samples. At high frequencies, the impedance of the chit/EE100 sample was the highest, which proved its higher corrosion resistance compared to others. However, for the lower frequency range, samples with coatings containing AgNPs showed higher impedance. The impedance value of the chit/EE100/AgNPs coated samples were comparable to the uncoated Ti grade 2 sample.

Table 6 compares the simulated test results of the samples using equivalent electrical circuits (inserted in Figure 8c: (a)—for bare Ti grade 2 substrate, (b)—for prepared coatings). The χ^2^ value remained at the level of 10^−3^ and 10^−4^, which proved a satisfactory fitting of the obtained results and the proposed equivalent circuit. Chitosan- and Eudragit-based coatings on the titanium surface increased the total resistance of samples. Moreover, the addition of Ag nanoparticles further increased the total resistance of the system. The EIS results were aligned with those obtained from the potentiodynamic method. According to Oliveira et al. [36], the corrosion-protective mechanism of chitosan-based coatings may be attributed to chemisorption occurring when the N and O heteroatoms present in the chitosan structure donate their free electron pairs to the empty d-orbitals of the metal surface and hence play the role of a physical barrier separating the titanium surface from the corrosion medium [37]. The incorporation of inorganic species, such as metal nanoparticles, into the biopolymer matrix can contribute to the increase of the protective effect. The implementation of AgNPs into the chit/EE100 matrix can enhance its adsorption process due to the direct interaction between the nanoparticles and the metal surface and the formation of a passive corrosion-resistant layer, boosting the barrier effect of the composite coating [69].

**Figure 9:**
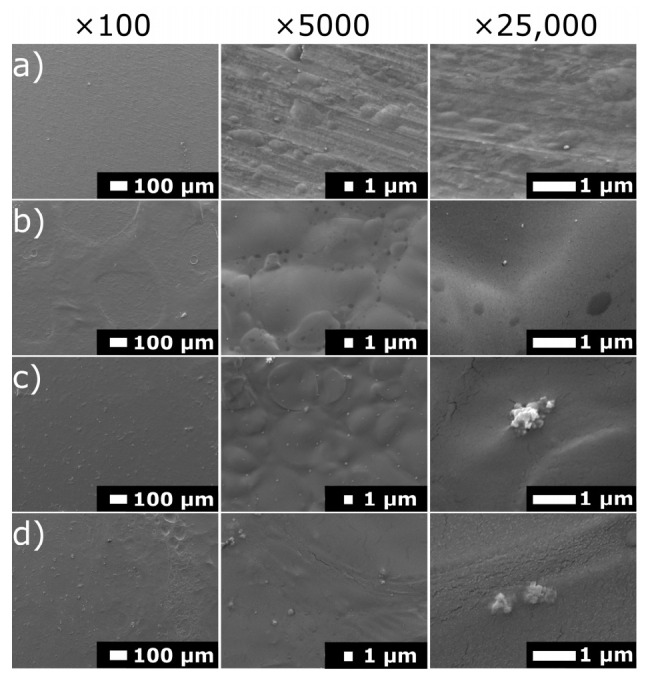
SEM images of the surface of the: (**a**) bare titanium substrate, (**b**) chit/EE100, (**c**) A1, and (**d**) B1 coatings after electrochemical tests; at different magnifications, ×100 (on the left), ×5000 (in the middle), and ×25,000 (on the right).

SEM images of the samples after electrochemical investigations are shown in Figure 9. In the case of Ti grade 2, no corrosion signs were noticed on the sample surface. In the case of coated samples, they swelled under the influence of staying in the simulated body fluid. Chit/EE100 Ag-doped coatings exhibited stability in a pH-neutral environment; hence, the silver nanoparticles were not released into the solution and could be recognized in the SEM images [27].

### 3.5. Silver Release Study

**Figure 10:**
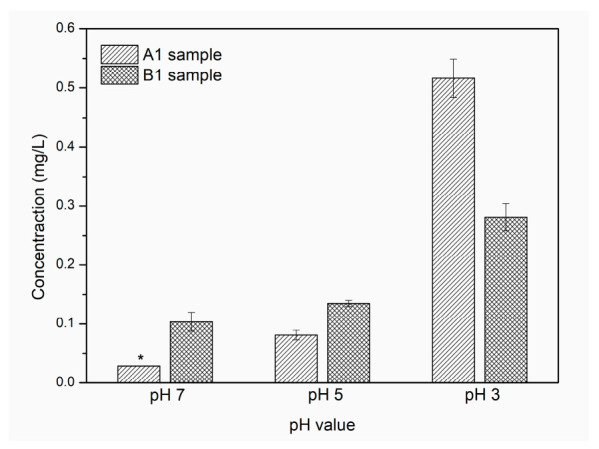
Cumulative concentrations of Ag ions released from chit/EE100/AgNPs coatings (A1 and B1 samples) after 1 day of exposure in SBF solution of different pH values at 39 °C. Data were reported as mean ± SD (*n* = 8); * the measured value was below the LOD (limit of detection) < 0.013 mg/L.

The silver release profiles from the chit/EE100/AgNPs coatings (A1 and B1 samples) after 1 day of exposure to simulated body fluid at different pHs (7, 5, and 3) are shown in Figure 10. It was observed that the concentration of Ag ions in the SBF solution increased as the pH value of the solution decreased. For neutral pH, the amount of silver released was much lower; for the A1 sample, the measured value was below the detection range of the measuring device, suggesting that this coating exhibited high stability in a neutral pH environment. When the pH value of the SBF decreased to simulate inflammation, there was a rapid release of silver ions. This was due to the dissolution process of the biopolymer matrix in which the silver nanoparticles were dispersed. The protonation of the amino groups of chitosan and EE100 is enhanced under lowered pH, and the repulsive interaction causes degradation of the biopolymer coating and release of silver nanoparticles [11,70]. Comparing the different silver content in the coatings, sample B1 exhibited a release of higher amounts of silver at pH 7 and 5 compared to sample A1, but at the lowest pH value, a sharp increase in Ag ion concentration was observed for sample A1, significantly exceeding the Ag ion content released from sample B1. According to the SEM and EDS results, this was possible because the silver nanoparticles in sample A1 showed a higher tendency to agglomerate; hence, at low pH, more large silver agglomerates were released, resulting in a higher Ag concentration in the SBF solution.

The minimum requirement for antibacterial activity of silver is at concentrations of at least 0.1 ppb [71]. In addition, the dose of allowable silver oral exposure determined by the United States Environmental Protection Agency (US EPA) equals 0.005 mg Ag/kg body weight daily. The results indicated that at a pH of 3 for sample A1, this value was slightly exceeded; however, for the B1 sample, the silver concentration was within limits [33].

Both coatings showed strong sensitivity to decreasing pH and could provide a system for controlled delivery of an antimicrobial substance to peri-implant tissues. This type of system would create a controlled release of the therapeutic substance at the onset of a local inflammation while maintaining high stability in the neutral state [72]. The high stability of chit/EE100/AgNPs composite coatings at neutral pH would reduce the risk of the adverse burst release phenomenon in the initial phase after implantation of such a biomaterial [73]. The duration of the study of silver release from chit/EE100/AgNPs coatings in SBF solutions with suitably modified pH was limited to 1 day because after this time in acidic pH, the composite coating was almost completely dissolved; hence, a longer study time would have been pointless. The main concern was to demonstrate the sensitivity of the produced coatings to a change in the pH of the media into which the samples would be introduced. Comparing the results obtained in the previous study [33], it can be said that the addition of Eudragit E 100 contributes to a significant reduction in chitosan coating degradation at neutral pH while maintaining high sensitivity to pH decrease, which is crucial in drug delivery control systems. Thus, this type of multilayer surface modification of the implant can provide long-term protection against biofilm formation and the development of postoperative infections, considered as one of the predominant causes of unsuccessful implant procedures [74].

## 4. Conclusions

Composite coatings of chit/EE100/AgNPs were successfully deposited on titanium substrates by one-step electrophoretic deposition. The applied process parameters significantly affect the morphology of the coatings formed, thus affecting their physical, mechanical, and electrochemical properties. Coatings with higher uniformity were obtained at the lower process values, i.e., voltage and deposition time.

The proposed system based on chitosan and Eudragit E 100 with the addition of silver nanoparticles exhibited good corrosion resistance, adequate wettability, and demonstrated strong sensitivity to a reduced pH environment, which is crucial for applications in controlled drug delivery systems.

The adhesion of the coatings to the metallic substrate was a short break of developed coatings. The addition of Eudragit E 100 did not significantly increase the mechanical properties of the chitosan coatings. Another disadvantage was the tendency of silver nanoparticles to form agglomerates, despite the addition of a dispersing agent.

To overcome these obstacles, future research should focus on improving coating adhesion, e.g., by modifying the method of preparing the metallic substrate before deposition of composite coatings. Agglomeration of silver nanoparticles can be prevented by adding more dispersing agents and modifying the preparation procedure of the EPD suspension.

## Figures and Tables

**Table 1:** The elemental composition of the substrate material, wt.%.

Element	H	N	C	Fe	O	Ti
wt.%	<0.001	<0.009	<0.013	0.168–0.179	0.170–0.190	rest

**Table 2:** Characteristics of the process parameters for particular test samples.

Sample	Suspension		Voltage (V)	Time (min)
A1	A (0.005 g AgNPs)	100 mL of 1 vol.% acetic acid, 0.1 mL of Polysorbate 20, 0.25 g of Eudragit E 100, and 0.1 g of chitosan	10	1
A3				3
A1′			30	1
A3′				3
B1	B (0.01 g AgNPs)		10	1
B3				3
B1′			30	1
B3′				3

**Table 3:** Surface topography parameters and coatings thickness of the studied samples.

Sample	Sa (nm)	Sp (nm)	Sv (nm)	Coating Thickness (µm)
Ti grade 2	142 ± 19	921 ± 310	−738 ± 158	-
Chit/EE100	81 ± 14	575 ± 167	−446 ± 141	3.61 ± 0.75
A1	82 ± 7	931 ± 416	−373 ± 90	2.69 ± 0.97
A3	99 ± 14	1065 ± 478	−415 ± 77	15.65 ± 2.29
A1′	148 ± 35	957 ± 128	−465 ± 122	13.88 ± 2.32
A3′	199 ± 17	1582 ± 346	−859 ± 41	31.79 ± 1.96
B1	91 ± 30	749 ± 230	−393 ± 128	2.65 ± 0.89
B3	86 ± 9	805 ± 378	−494 ± 228	13.09 ± 1.73
B1′	200 ± 34	1223 ± 170	−896 ± 68	10.47 ± 1.05
B3′	156 ± 9	812 ± 64	−729 ± 47	15.65 ± 1.50

**Table 4:** Scratch test properties of the chit/EE100 and chit/EE100/AgNPs coatings (mean ± SD; *n* = 10).

Sample	Critical Load, *Lc* (mN)	Critical Friction, *Lf* (mN)
Chit/EE100	32.58 ± 14.52	35.70 ± 19.03
A1	51.76 ± 13.53	20.31 ± 11.40
A3	24.95 ± 9.12	49.96 ± 31.01
A1′	27.58 ± 8.22	46.89 ± 18.07
A3′	34.53 ± 12.04	54.84 ± 29.41
B1	40.22 ± 9.17	27.61 ± 9.68
B3	27.96 ± 8.74	30.24 ± 26.11
B1′	18.69 ± 10.03	33.39 ± 10.57
B3′	32.10 ± 10.29	31.41 ± 17.40

**Table 5:** OCP, E_corr_, and i_corr_ values of the bare titanium substrate, chit/EE100 coating, and chit/EE100/AgNPs coatings (A1 and B1 samples).

Sample	OCP (V)	E_corr_ (V)	i_corr_ (nA/cm^2^)
Ti grade 2	−0.298	−0.464	190.55
Chit/EE100	−0.162	−0.348	177.83
A1	−0.108	−0.371	169.82
B1	−0.092	−0.338	158.49

**Table 6:** Simulated parameters of EIS data using the proposed equivalent circuits for the investigated samples.

Sample	R_s_ (Ωcm^2^)	CPE_1_-T (µFcm^−2^)	CPE_1_-P	R_1_ (Ωcm^2^)	CPE_2_-T (µFcm^−2^)	CPE_2_-P	R_2_ (MΩcm^2^)	CPE_3_-T (µFcm^−2^)	CPE_3_-P	R_3_ (Ωcm^2^)	χ^2^
Ti grade 2	35.07	1.261	0.84	12.87	-	-	-	-	-	-	0.008665
Chit/EE100	34.18	0.799	0.81	16.38	26.35	0.90	6.91	141.38	0.67	100.20	0.000398
A1	34.20	0.299	0.86	14.89	25.06	0.95	7.27	85.57	0.92	3.98	0.004804
B1	29.20	0.950	0.78	17.95	21.60	0.96	7.89	7030.00	0.99	50.10	0.005371

## Data Availability

The data presented in this study are available on request from the corresponding author.
